# The impact of age and body weight on acclimation to electronic feeding stations of weaning lambs

**DOI:** 10.1016/j.vas.2026.100576

**Published:** 2026-01-16

**Authors:** Henrietta Nagyné Kiszlinger, Miklós Szabari, György Kövér

**Affiliations:** Hungarian University of Agriculture and Life Sciences - Kaposvár Campus Hungary.

**Keywords:** Electronic feeder, Automatic feeder, Weaned lambs, Adaptation, Feeder visit

## Abstract

The use of electronic and automatic feeders has become widespread in modern livestock farming for gathering precise data on individual animals. While these technologies are commonly used for pigs and dairy cattle, there is very limited information on their application in sheep. Specifically, the time it takes for lambs to acclimate to these new feeding stations remains largely unknown. The use of electronic feeders equipped with entry-exit gates was studied with weaned lambs. 36 Suffolk lambs were observed over a 48-hour period. Data on the first visit of the feeders and the frequency of visits were collected over a 48-hour period. Additionally, the feeder’s regular use, defined as visiting the feeder station for three consecutive days, was evaluated over an extended period. The animals were provided additional feed in troughs until the end of the second day of the study. All lambs (100 %) adapted to the electronic feeder without prior training within two days. Key findings revealed no significant difference between sexes in the time taken for the first feeder visit. However, the age (*P* = 0.048) and body weight (*P* = 0.035) of the lambs significantly influenced the initial visitation time, suggesting that younger/lighter individuals may approach the novel system sooner. Furthermore, 72.2 % of the lambs established regular feeder use from the first day. Further study is recommended to determine the extent to which the adaptation period is shortened without supplementary feed.

## Highlights

• Lambs adapt to the use of electronic feeder without prior training within two days.
• The age and weight of the lambs influences the first time of feeder visit.
• The majority of the lambs establish a regular feeder visit from the first day.

## 1 Introduction

The use of electronic or automatic feeders has become widespread nowadays in commercial livestock farming. Electronic feeders enable precise feeding control, allowing farmers to adjust feed rations to the specific nutritional needs of individual animals or groups (Zhao et al., 2019). These systems are widely adopted in the swine and dairy industries, where various technologies exist for individual animal management (Zhao et al., 2019). However, the application of this technology to sheep has not been widely explored. This is primarily due to keeping sheep mostly under extensive conditions (Jorgensen & Boe, 2015). As a result, there is a significant lack of published research on the adaptation of sheep to electronic feeders. Specifically, there is little to no information on the training methods or the necessary acclimation period for lambs (Jorgensen & Boe, 2015; Vallière et al., 2022), that access feed in a way that is basically different from traditional grazing or trough feeding.

This study was designed to fill this gap. The novelty of our approach lies in quantifying the speed of behavioral acclimation to complex electronic feeding stations in young lambs, a population for which adaptation data is currently unavailable. We investigated lambs' first feeder visit, the frequency of feeder visits, their potential sex differences, and the development of regular use of the electronic feeders. The findings of this research will provide a foundation for implementing this technology in sheep farming, ensuring that animal welfare is maintained while improving the precision of farm management and performance testing.

## 2 Materials and methods

The study was conducted at the training farm of the Hungarian University of Agriculture and Life Sciences, Kaposvár Campus (Kaposvár, Hungary), in June of 2024. A total of 36 Suffolk lambs, comprising 21 females and 15 males, were included in the study. The lambs' ages ranged from 39 to 80 days, and their weights ranged from 20 kg to 47.5 kg. Categorized by sex, the males' age and weight averaged 71.8 ± 3.8 days and 32.2 ± 6.4 kg, respectively, while the females' age and weight averaged 70.7 ± 8.7 days and 31.3 ± 5.7 kg, respectively. The wide variability in age and weight among the lambs is attributable to the standard weaning protocol of the training farm, wherein all lambs were weaned simultaneously, irrespective of individual differences in age or weight. We included all lambs born during the study period. The study is out of the scope of the 2010/63/EU Directive, but is carried out with the knowledge and permission of the Institutional Animal Welfare Committee of Hungarian University of Agricultural and Life Sciences Kaposvár Campus (Reg. No. MATE KC MÁB 2024/10/2).

### 2.1 Housing and feeding

The newborn lambs were isolated from the main flock along with their dams immediately after birth for a period of 10 days to allow the mother to bond with her lamb. Following this period, both the lambs and their dams were reintroduced to the flock in their original pen, and from that point onward the lambs were provided with unrestricted access to pelleted starter feed. Weaning was conducted simultaneously for all lambs, with the age corresponding to the age range specified above. The lambs were moved to the research unit of the training farm.

Then the lambs were allocated to the five experimental groups based on sex (because we were concerned that the more assertive behavior of the male animals could influence the feeding behavior of the females), with three pens designated for females and two pens for males, using simple randomization. This process was executed by a stockperson who was blinded to the study's objectives. Each pen, measuring 4 × 4 m, was equipped with straw bedding. Since each pen housed only one sex, there is a potential confounding between sex and pen effects.

All lambs were marked with RFID ear tags for identification. Natural light was provided through windows, following the natural photoperiod in June and the outdoor conditions. Average daily temperatures during the study period ranged from 20,3 to 26,1 °C, and relative humidity ranged from 65 to 80 %. The meteorological data were obtained from the database of the Hungarian Meteorological Service (HungaroMet). Specifically, we used the datasets from the Kaposvár station. The average background noise level in the barn was measured at 55 dB (dB). The sound of the electronic feeder opening was recorded at 74 dB.

On the first day, the animals had unrestricted access to the previously introduced pelleted starter lamb feed in troughs, hay in hayracks, and water from automatic cup drinkers, while the electronic feeder stations remained closed. After a 24-hour adaptation period to the new housing environment, the electronic feeders were activated at 8:00 am (1st day of the study). The lambs had free access to a portion of feed at any time of the day, with a maximum limit of 2 kg of feed per day. During this time, the lambs also continued to have access to the feed in the troughs. As the time required for the lambs to learn to use the feeder consistently was uncertain, we opted not to withhold their usual pelleted feed in the troughs to prevent any setback in the development of those intended for breeding. From the third day onward, no additional feed was provided other than through the electronic feeders.

### 2.2 Electronic feed station

**Photo 1:**
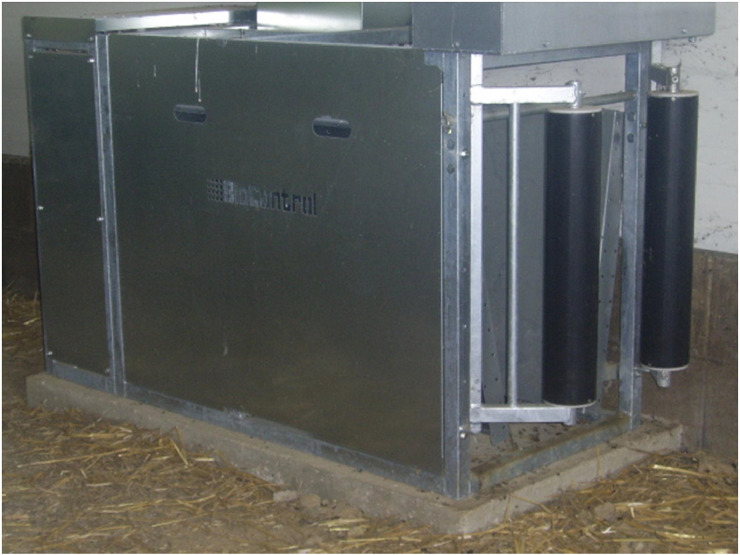
SF61 electronic sheep feeder. The photo shows two pneumatically operated wings of the entrance to the feeding station.

The electronic feeding stations (Photo 1.) were made by the Norwegian company Biocontrol AS. In its default state, the electronic feeder is open when not in use by any lamb. When an animal entered the station, the transponder was read as soon as it reached in the antenna field at the feed bowl and a portion of the daily feed ration was dispensed. At the same time the pneumatic entry gate closed. If the animal left the one-way feeder station, the entry gate opened again after the “gate time out”, that is, when no transponder was present in the antenna field. In this case, we set it to 10 s.

Evaluation of the learning behaviour: the time when the ear tag of the animals was first registered in the feeder’s software was analysed and also compared between the sexes in 24 h. We evaluated it in relation to age and weight of the lambs at weaning, which were included in the statistical model. All subsequent times of feeder visits were automatically registered in the feeder’s software when the lambs entered the feeding station.

### 2.3 Statistical analysis

All statistical analyses were performed using SAS software (Copyright © 2024 SAS Institute Inc.) with a significance level set at 5 %. To evaluate the impact of sex on the time taken for lambs to learn to use the feeder in the first 24 h, we conducted a survival analysis using the Cox proportional hazards model. To address the potential pseudo replication due to having multiple individuals per pen, which results in clustering by pens, we employed a frailty model, which incorporates a random effect associated with the clustering variable (pen).

### 2.4 We specified the Cox model as

$$h_{ij}\left(t\right)=h0\left(t\right)exp\left(\beta 1\cdot sex_{i}+\beta 2\cdot weight_{ij}+\beta 3\cdot age_{ij}+u_{j}\right)$$

where:

- h_ij_(t) is the hazard function for the i^th^ lamb in the j^th^ cluster (pen) at time t.
- h0(t) is the baseline hazard function, which is common to all lambs
- sex_i_, weight_ij_ and age_ij_ are the covariates (sex, weight, and age, respectively) for the i^th^ lamb in the j^th^ pen
- β1, β2, and β3 are the coefficients corresponding to the covariates
- u_j_ is the frailty term, which represents the random effect associated with the j^th^ pen

We assessed multicollinearity between the predictors in the model (weight and age) using Variance Inflation Factor (VIF). The VIF values for both predictors were below 5 (1.5 for both) and correlation between them was moderately high (*r* = 0.55), indicating that multicollinearity is not a concern. Thus, weight and age contribute independently to the model.

The visit counts and their distribution in the first 6 and 24 h were compared with the Wilcoxon rank-sum test. Also, the effect of the weight on the regular use (defined as visiting the feeding station for three consecutive days) of the feeders was evaluated by dividing the lambs into two groups: those with regular feeder use from the first day, and those who started later. The two groups were then compared using an independent samples *t*-test.

## 3 Results

**Fig. 1:**
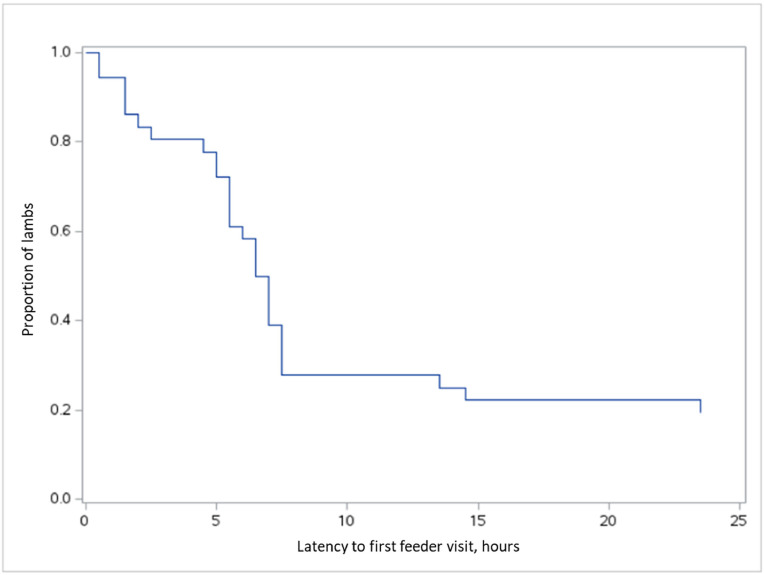
Survival curve of lambs showing the latency to their first feeder visit. The x-axis represents the time in hours, and the y-axis shows the proportion of lambs that had not yet visited the feeder. The curve indicates that the majority of first visits occurred within the first few hours, and by 24 h, 19.4 % of the lambs had not yet visited the feeder.

Within the first 24 h, 72.2 % of lambs successfully started to use the feeders, with the majority of these visits occurring within 7.5 h. By the end of the observation period, 19.4 % of lambs had not yet made a first visit (Fig. 1.). Those lambs that did not enter the station within the initial 24 h completed the task in the final hour of 48 h.

**Fig. 2:**
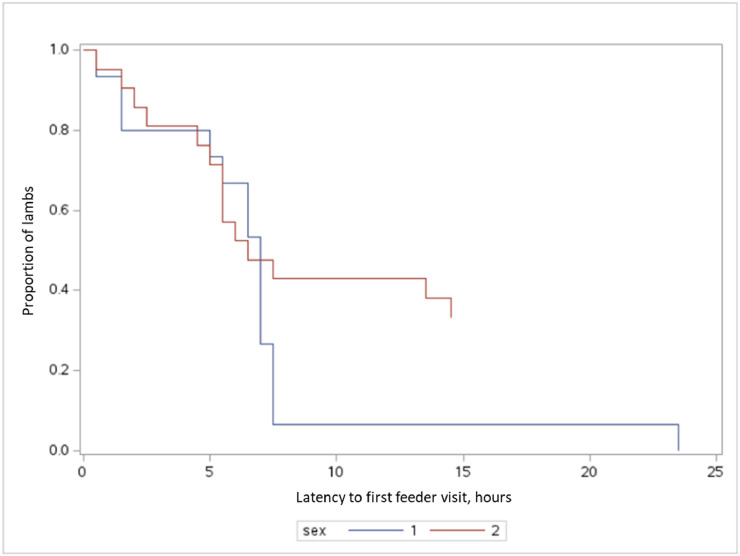
Survival curve of lambs showing the latency to first feeder visit, stratified by sex. The x-axis represents time in hours, and the y-axis shows the proportion of lambs that had not yet visited the feeder. Male lambs (blue line) showed a more rapid decrease in latency compared to female lambs (red line), indicating they learned to use the feeder more quickly.

Males demonstrated a faster adaptation to the feeders than females (Fig. 2). While the first visit was recorded by a male lamb just 3 min after the stations were activated, the difference between sexes was most evident at the 7.5-hour mark. At this point, nearly all males (93.3 %) had made their first visit, compared to only 57.1 % of females. Seven females did not explore the feeder within the initial 24 h, but all lambs eventually learned to use the station within the 48-hour follow-up period. In this comparison, we have not yet taken into account the effects of weight and age.

**Fig. 3:**
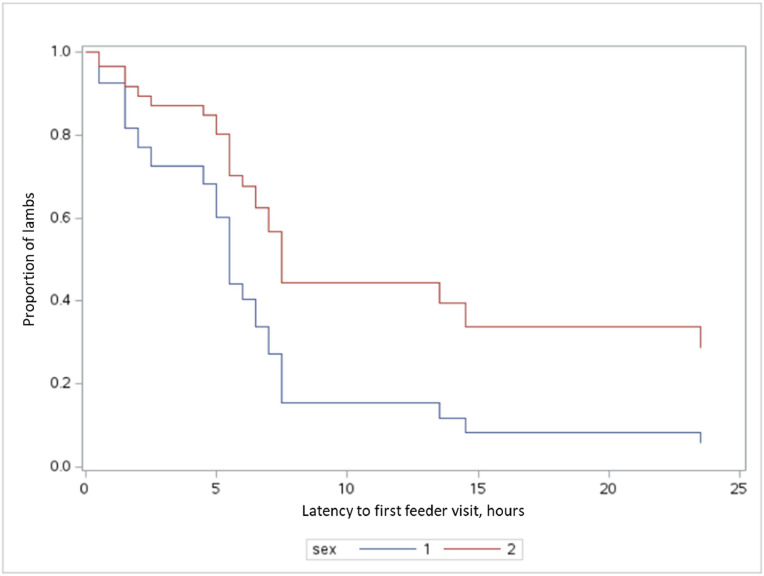
Survival curve of lambs by sex, generated from a Cox model without random effects. The curves show the adjusted latency to first feeder visit. This visualization was created to display the effects of fixed variables (sex, age, body weight) on survival, separate from the pen-specific random effects.

The outcome of the Cox proportional hazards frailty model revealed that while sex did not significantly influence a lamb's time to first feeder visit (*P*= 0.45), both age and body weight had a significant, albeit small, negative effect. As a lamb's age increased, its likelihood of visiting the feeder in a given time period decreased slightly (hazard ratio = 0.94, 95 % CI: 0.88, 1.00). Similarly, a higher body weight was associated with a slightly reduced likelihood of a timely first visit (hazard ratio = 0.89, 95 % CI: 0.79, 0.99). This analysis, which included a random effect to account for the variation in learning times among different pens, indicates that older and heavier lambs were moderately slower to begin using the feeder. Fig. 3. was generated based on the Cox model without the inclusion of the random effects in the plot itself. This approach was used to simplify visualization and ensure that survival curves for the fixed effects could be plotted clearly.

**Fig. 4:**
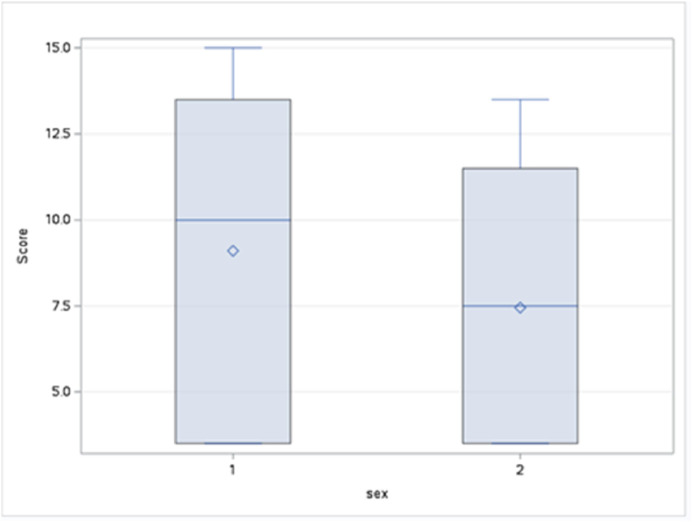
Distribution of Wilcoxon scores for visit counts by sex (first 6 h). The boxplots show the distribution of Wilcoxon scores for visit counts during the first 6 h. While not statistically significant, the data suggest that males had a higher median score than females, indicating a tendency for more visits. The greater interquartile range for males also suggests more variability in their visit counts compared to females.

**Fig. 5:**
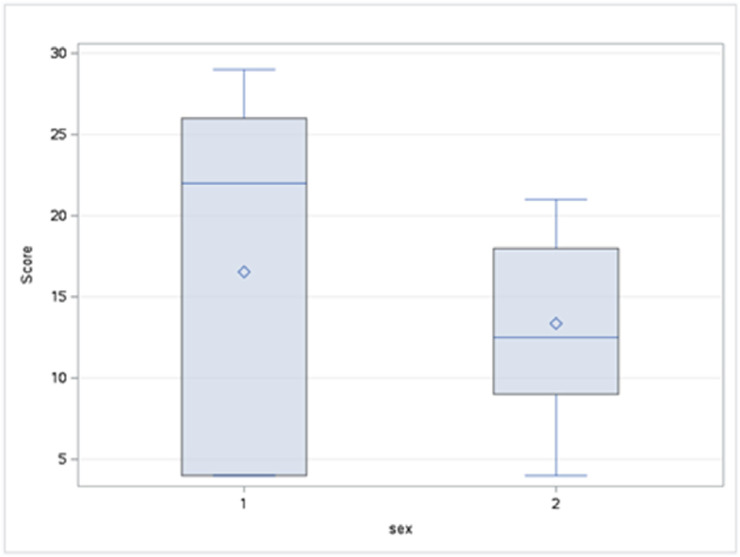
Distribution of Wilcoxon scores for visit counts by sex (24-hour study). The boxplots display the distribution of Wilcoxon scores for visit counts over 24 h. The data show that while not statistically significant, males had a higher median score than females. The larger interquartile range for males also indicates greater variability in visit counts compared to females.

The visit counts were compared between the sexes using the Wilcoxon two-sample test due to non-normal data distribution. The analysis showed no significant differences in visit counts between the sexes, neither for the first 6 h (*P* = 0.5) (Fig. 4.) nor for the full 24 h (*P* = 0.32) (Fig. 5.) of the study. Despite the lack of statistical significance, males tended to have a higher mean Wilcoxon score for both time periods.

**Fig. 6:**
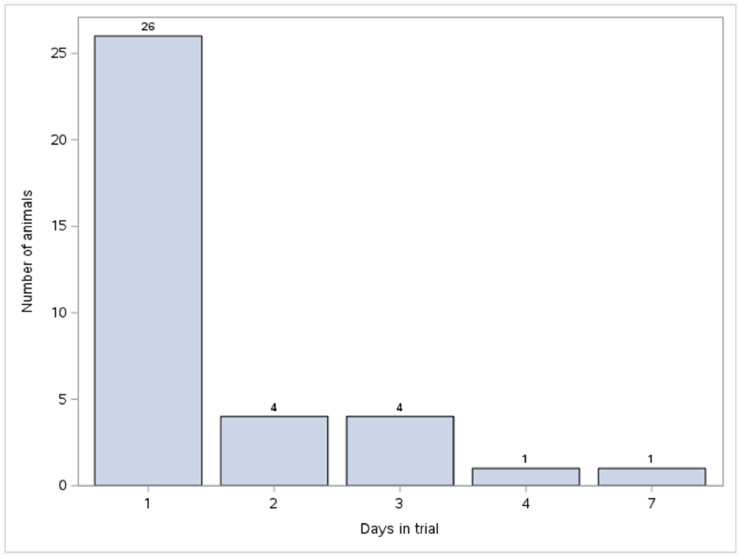
Number of animals that started regular feeder visits. The bar chart shows the number of animals that began consistently visiting the feeders for at least three consecutive days, categorized by the day they started. Most animals (26 of 36) initiated regular visits on the first day of the trial, while the remaining animals transitioned to regular feeder use by day 7.

Most animals quickly established a routine; 72.2 % (26 of 36) met the criteria for regular feeder use (visiting on three consecutive days) starting from the first day (Fig. 6). Following the removal of ad libitum feed on day 3, all but two of the remaining animals transitioned to regular feeder use. The one that developed regular feeder visit on day 4 was in the middle range in terms of weight, while the one that started using the feeder regularly on day 7 was the second heaviest lamb. Both were in the middle range in terms of age. The importance of weight was again confirmed. Lambs that used the feeder regularly from day one had significantly lower weight (*P* = 0.048) than those who started later.

## 4 Discussion

### 4.1 Feeder adaptation

Transitioning from the conventional feeding systems where the feed is visible in the troughs to automatic feeding stations where, on the contrary, the animals do not see the feed before entering the station and reading the RFID ear tag may raise the question of how long it takes for the animals to learn using a more complex system.

The rapid adaptation of lambs to the electronic feeders is a key finding of our study. The majority of animals (72.2 %) began regular use on the very first day, and all animals visited the feeder at least once within 48 h. This swift learning suggests that with the tested type of feeder, lambs do not require extensive human intervention or training, which has significant practical implications for sheep farming. This result is supported by David et al. (2014), who observed that lambs (5–28 days old) adapted to an automatic milk feeder, designed with a narrow corridor for single access, within one day. Our findings are particularly noteworthy when compared to the study by Jorgensen and Boe (2015). In their experiment with a feeder of similar design using adult Dala ewes, they reported slower adaptation rates, with some ewes not adapting even after 5 days. This contrast suggests that the difference in learning speed is not solely due to the physical attributes of the feeder, such as the clear line of sight or the presence of a pneumatic gate. Instead, the rapid adaptation in our study may be attributed to other factors, such as the age of the animals or breed-specific behavioral differences, or the management practice of creep feeding. Since our lambs were already habituated to seeing feeders in a 'lamb nursery' environment, their transition to the automated station was likely a task of cognitive adaptation to the gate mechanism rather than a primary learning of where the food is located. However, it is important to note that initial hesitation is also observed in other species: Wilson et al. (2018) found that the latency to the first visit for calves using automatic milk feeders (AMF) was approximately 15.13±12.99 in solid-side stall design and 17.31±14.28 in steel-bar stall design hours after initial training, highlighting the cross-species variability in adaptation time. Furthermore, Valliére et al. (2022) found that post-weaning lambs, when provided with their standard trough feeders for 10 days alongside the new automatic SmartFeeder, only showed a daily use rate between 64 % and 78 % after 6 days. This suggests that the continued availability of familiar feeding resources may reduce the motivation for rapid adaptation to the new system.

Our results indicate that young lambs exhibit rapid habituation to the mechanical components of the feeder, such as the pneumatic gate, suggesting high behavioral flexibility in a novel feeding environment.

### 4.2 Factors influencing adaptation: behavior, age and sex

While our results suggest that most lambs adapt quickly, certain lambs show a delayed response. The underlying reasons are likely behavioral and rooted in individual temperament. Coulon et al. (2015) found that prenatally-stressed lambs were characterized by a negative affective state, increased fear reactions, and impaired cognitive evaluation, illustrating the long-lasting impact of early life conditions on coping strategies. Similarly, Bickell et al. (2011) suggested that "calm" and "nervous" lambs may adopt different coping styles when exposed to a challenging (noisy, mobile) novel object.

Our data indicate that younger animals possess a significantly higher capacity for learning new feeding behaviors. This heightened behavioral plasticity in younger lambs is consistent with the findings of Sena et al. (2013) who observed better learning and memory capability and lower stress behavior in lambs weaned at 4 weeks compared to 6 or 8 weeks, and also Mialon et al. (2023) indicated that younger animals may have a higher capacity for learning new feeding behaviors. This is a reasonable behavioral mechanism, as younger animals are often more curious and less set in their ways than older ones (Glickman & Sroges, 1966).

However, the literature presents contradictory findings regarding the effect of age on learning. For instance, in a study analyzing spatial learning in a T-maze, Johnson et al. (2012) found that 14-week-old lambs learned the task significantly faster than 9-week-old lambs. This contradiction suggests that the benefit of younger age may be context-dependent or related to specific developmental milestones. Additionally, Viérin et al. (2003) assessed the effects of age on fear reactions (isolation, surprise, human presence) and found that 3- to 4-month-old lambs were more fearful than 5- to 6-month-old lambs. Also Jorgensen and Boe (2015) found different results in young sheep. While one group learned the task within 6 h, some individuals in another group needed up to three days. This highlights the complex relationship between age, fear (which could affect approach to the pneumatic gate), and learning capacity.

The unexpected noise made by the air-pressure entrance gate may contribute to the animals’ initial fear and so it could influence the learning process. A study by Vandenheede and Bouissou (1993), which investigated fear reactions in sheep, described a similar situation where the sudden noise of a gate closing could be comparable to a ball dropping from the ceiling. Confirming this tendency, Robinson et al. (2014) found that males are more likely to leave their conspecifics to approach a novel object than females, even when the object is mobile and noisy. Viérin et al. (2003) also observed that females were more fearful than males when submitted to fear-eliciting situations. Pérez-Barbería et al. (2004) found that males may be less fearful and demonstrate greater perseverance compared to females when accessing food items in novel or challenging feeding stations. However, these sex differences primarily relate to fear and approach behaviour, not necessarily cognitive ability. Johnson et al. (2012), using a T-maze task, found no main or interactive effects of sex on spatial learning, suggesting that while males may be more motivated or quicker to overcome fear barriers, the underlying learning capacity may be similar across sexes. This contrast between differences in motivation/fear and learning ability requires further clarification.

### 4.3 Welfare considerations and practical implications for sheep farming

Our findings have some important implications for sheep producers. As demonstrated by our results, the rapid and autonomous adaptation of lambs to electronic feeders means that human labor for training can be minimized or eliminated. While traditional feeding systems serve as a baseline for basic nutrition, the primary strength of automated systems lies in their capacity for high-resolution data collection and precision monitoring of individual feeding patterns. However, the validity and utility of such data are entirely dependent on the animals' consistent and stable use of the device. Therefore, assessing the speed of adaptation is a critical prerequisite for the successful implementation of precision livestock farming (PLF) technologies, making the study of learning latency a priority over direct comparisons with conventional feeding methods**.** The use of automated feeders also offers significant welfare benefits. Rushen and Passille (2012) highlight that automated feeders (e.g., for calves) can help measure the degree that animals are hungry and have the potential to identify sick animals early even in group housing by monitoring individual feeding patterns. Furthermore, proper feeder design can mitigate negative welfare outcomes. Mohapatra et al. (2024) demonstrated that conventional feeders (without dividers) resulted in significantly lower eating time and stronger agonistic behavior (intense competition), leading to minimal weight gain and maximal feed wastage. By contrast, the automated system evaluated in our study provides a solution to these welfare and economic challenges by ensuring individualized access without the need for constant human supervision. With individual feeding data, producers can monitor daily feed intake and adjust rations for each animal (Zhao et al. 2019). Due to the quick transition, the risk of a significant period of underfeeding is low. Understanding this speed of adaptation is crucial for optimizing lamb fattening systems, as it allows producers to strategically time the introduction of automated technologies to minimize environmental stress. This is particularly crucial for young, growing lambs.

### 4.4 Study limitations and future directions

Since each pen housed only one sex, there is a potential confounding between sex and pen effects. Although the pens were identical in setup, subtle environmental or social factors unique to each pen could still influence the results. Furthermore, the relatively small sample size and the specific management background of the studied lambs (i.e. pre-exposure to creep feeding) may influence the generalizability of our findings to regions with different early-life management practices. Caution should be used when applying these results to settings where both male and female lambs are housed in the same pen, as behavioural and physiological interactions in mixed-sex pens could yield different outcomes. Additionally, it is important to acknowledge that our study focused exclusively on the temporal aspect of adaptation (time-to-event). While this provides a clear metric for learning speed, it does not account for individual variations in metabolic state or specific social motivations that might influence feeder attraction. Furthermore, as this study was designed to evaluate the adaptation process itself rather than long-term performance, the results should be interpreted within the context of early-stage technology introduction.

Future research should specifically address the limitations of this study by linking them to current research gaps. The contradictory results regarding the effect of age on learning, the contrast between sex differences in fear or approach, and the lack of sex difference in learning present key research gaps that need to be addressed. It is crucial to redesign the study to mitigate the sex-pen confounding issue, which can be done by housing mixed-sex groups in multiple pens. In addition, it is necessary to conduct trials with a larger sample size and different sheep breeds to confirm our findings and allow for broader generalization. Further study is also recommended to determine the extent to which the adaptation period is shortened when the lambs are introduced to the electronic feeder without supplementary feed.

## 5 Conclusions

Weaned lambs do not need human assistance to get used to the electronic feeder within two days. If the farm technology uses this feeding technique for post-weaning lamb rearing, it is advisable to transition the animals to the new feeding technology early and at a lower body weight. This should be only when the animals' independent feed intake is sufficient for their uninterrupted development. Due to the problem of confounding effects, only cautious conclusions can be drawn from the comparison of sexes. However, based on the results, no special attention needs to be paid to either sex. Further study is recommended to determine the extent to which the adaptation period is shortened without supplementary feed or by incorporating visual/olfactory attractants in the feeders. It would also be interesting to examine how different feeder settings (e.g., changes in feed allowance, timing) influence the number of times lambs visit the feeder. This could have an impact on feed intake and growth, providing insights for optimizing production.

## Funding

This research received no external funding.

## Institutional review board statement

Ethical review and approval were waived for this study due to the research being purely observational, and out of the scope of the 2010/63/EU Directive.

## Informed consent statement

Not applicable.

## Ethical statement

The study is out of the scope of the 2010/63/EU Directive, but it is carried out with the knowledge and permission of the Institutional Animal Welfare Committee of Hungarian University of Agricultural and Life Sciences Kaposvár Campus (Reg. No. MATE KC MÁB 2024/10/2).

## CRediT authorship contribution statement

**Henrietta Nagyné Kiszlinger:** Writing – original draft, Methodology, Investigation, Formal analysis, Data curation, Conceptualization. **Miklós Szabari:** Project administration. **György Kövér:** Validation, Supervision, Conceptualization.

## Declaration of competing interest

The authors declare that they have no known competing financial interests or personal relationships that could have appeared to influence the work reported in this paper.

## Data Availability

Data will be made available on request.
